# Synergistic infection of BrYV and PEMV 2 increases the accumulations of both BrYV and BrYV-derived siRNAs in *Nicotiana benthamiana*

**DOI:** 10.1038/srep45132

**Published:** 2017-03-27

**Authors:** Cui-Ji Zhou, Xiao-Yan Zhang, Song-Yu Liu, Ying Wang, Da-Wei Li, Jia-Lin Yu, Cheng-Gui Han

**Affiliations:** 1 State Key Laboratory for Agro-Biotechnology and Ministry of Agriculture Key Laboratory for Plant Pathology, China Agricultural University, Beijing, 100193, P. R. China

## Abstract

Viral synergism is caused by co-infection of two unrelated viruses, leading to more severe symptoms or increased titres of one or both viruses. Synergistic infection of phloem-restricted poleroviruses and umbraviruses has destructive effects on crop plants. The mechanism underlying this synergy remains elusive. In our study, synergism was observed in co-infections of a polerovirus Brassica yellows virus (BrYV) and an umbravirus *Pea enation mosaic virus 2* (PEMV 2) on *Nicotiana benthamiana*, which led to (1) increased titres of BrYV, (2) appearance of severe symptoms, (3) gain of mechanical transmission capacity of BrYV, (4) broader distribution of BrYV to non-vascular tissues. Besides, profiles of virus-derived small interfering RNAs (vsiRNAs) from BrYV and PEMV 2 in singly and doubly infected plants were obtained by small RNA deep sequencing. Our results showed that accumulation of BrYV vsiRNAs increased tremendously and ratio of positive to negative strand BrYV vsiRNAs differed between singly infected and co-infected plants. Positions to which the BrYV vsiRNAs mapped to the viral genome varied considerably during synergistic infection. Moreover, target genes of vsiRNAs were predicted and annotated. Our results revealed the synergistic characteristics during co-infection of BrYV and PEMV 2, and implied possible effects of synergism have on vsiRNAs.

RNA silencing, a highly conserved mechanism in various eukaryotic organisms, regulates gene expression and plays a major role in antiviral immunity^1^,^2^. A typical trait of antiviral silencing in plants is production of virus-derived small interfering RNAs (vsiRNAs)^3^,^4^,^5^. For positive-strand plant RNA viruses, vsiRNAs are processed either from highly structured regions of viral genome or double-stranded viral RNA replication intermediates by Dicer-like (DCL) proteins^6^,^7^. These vsiRNAs are recruited by ARGONAUTE (AGO) containing RNA induced silencing complex (RISC) to guide them to the complementary viral RNAs or host transcripts in a sequence-specific manner^2^,^8^,^9^,^10^. Biogenesis of vsiRNAs from plant RNA viruses is dominated by DCL4 and DCL2. DCL4 is the key protein in vsiRNAs production and generates 21 nt vsiRNAs. When DCL4 is absent, DCL2 acts as an alternate to produce 22 nt vsiRNAs, which are sufficient to trigger antiviral immunity^11^. The vsiRNAs processed directly from the infecting viral genome by DCL proteins are called primary vsiRNAs^2^,^4^. To trigger the efficient silencing, secondary vsiRNAs are produced by plant RNA-dependent RNA polymerase. In *Arabidopsis*, RDR1 and RDR6 are major players in the generation of secondary vsiRNAs^12^,^13^.

vsiRNAs play pivotal roles in plant antiviral defence^2^,^4^,^14^. VsiRNAs can trigger post-transcriptional gene silencing (PTGS) to downregulate viral RNA^4^,^14^. Furthermore, vsiRNAs can also mediate transcriptional gene silencing (TGS) through RNA directed DNA methylation pathway to defence against some plant DNA viruses^15^,^16^. Viruses encode different viral suppressors of RNA silencing (VSRs) to counter antiviral defence by targeting multiple steps of RNA silencing pathway and thus inhibit the vsiRNAs function^17^.

In addition, vsiRNAs may function to silence complementary host transcripts at post-transcriptional level. It is reported that vsiRNA derived from *Tobacco mosaic virus* Cg can down-regulate the *Arabidopsis* At2g16595 and At1g30460 mRNAs^18^. Moreover, mRNA of magnesium protoporphyrin chelatase subunit I (*ChlI*) can be silenced by vsiRNA from *Cucumber mosaic virus*-Y satellite RNA, thus resulting in yellowing symptom in *Nicotiana benthamiana*^9^,^10^. It is recently showed that some tomato *callose synthase* transcripts are down-regulated by vsiRNA generated from the virulence modulating region of *Potato spindle tuber viroid*^19^.

Viral synergism occurs commonly in nature and is caused by co-infection of two unrelated viruses in the same host, resulting in increased accumulation of one or both viruses and occasionally more severe symptoms^20^,^21^. A number of devastating crop diseases are the outcomes of viral synergism^22^,^23^,^24^. Viral synergism can alter viral traits such as virus accumulation^20^,^23^,^25^, tissue tropism^26^, host range^27^ and transmission rate^28^. There is accumulating evidence that interviral synergy can affect the characteristics of vsiRNAs derived from co-infecting viruses^25^,^29^. It has been reported that synergistic interaction between crinivirus *Sweet potato chlorotic stunt virus* (SPCSV) and begomovirus *Sweet potato leaf curl virus* isolate StV1 in sweet potato leads to specific changes in the relative abundance and distribution of SPCSV-derived siRNAs, implying a distinctive influence of begomoviruses on RNA silencing of SPCSV in synergy^29^.

*Polerovirus* is one of the most economically important taxa of plant viruses^30^. Synergistic interactions between poleroviruses and umbraviruses have destructive effects on crop plants^24^,^31^. Poleroviruses are restricted to host phloem tissue^32^,^33^ and cannot be transmitted mechanically^34^.

It has been demonstrated that *N. benthamiana* plants co-infected with polerovirus *Potato leafroll virus* (PLRV) and umbravirus *Pea enation mosaic virus 2* (PEMV 2) show severe symptoms^26^,^35^. Besides, PEMV 2 helps PLRV to move out of the phloem into mesophyll tissues and transmitted mechanically^26^,^35^. It is also suggested that both cell-to-cell movement function and ability to overcome RNA silencing are important in mechanical transmission of PLRV^26^. VsiRNAs are vital in RNA silencing mediated antiviral defence^4^,^14^. However, possible roles of vsiRNAs in the synergistic interaction of poleroviruses and umbraviruses is poorly understood.

Brassica yellows virus (BrYV, a provisional name) is a tentative polerovirus that distributes in China, South Korea, and Japan^36^,^37^,^38^,^39^. BrYV infects a wide range of cruciferous crop plants^36^ and tobacco^40^. Co-infection of BrYV and umbraviruses in nature is not reported. In this study, we found that co-infection of BrYV and PEMV 2 produced severe symptoms and increased the accumulation of BrYV RNAs and CP in *N. benthamiana* plants. BrYV can be mechanically transmitted and invaded non-vascular tissues when co-infected with PEMV 2. To study the characteristics of vsiRNAs produced in co-infection of poleroviruses and umbraviruses, we further profiled vsiRNAs from BrYV and PEMV 2 in singly and doubly infected plants by small RNA deep sequencing, respectively. Synergistic infection of BrYV and PEMV 2 increased the accumulation of BrYV vsiRNAs and altered the ratio of positive to negative strand BrYV vsiRNAs. Moreover, co-infection dramatically changed the relative amounts and positions to which the BrYV vsiRNAs mapped to the virus genome. Target genes of some vsiRNAs derived from BrYV and PEMV 2 were predicted and annotated.

## Results

### Co-infection of BrYV and PEMV 2 produced severe symptoms and increased the accumulation of BrYV in *N. benthamiana*

**Figure 1:**
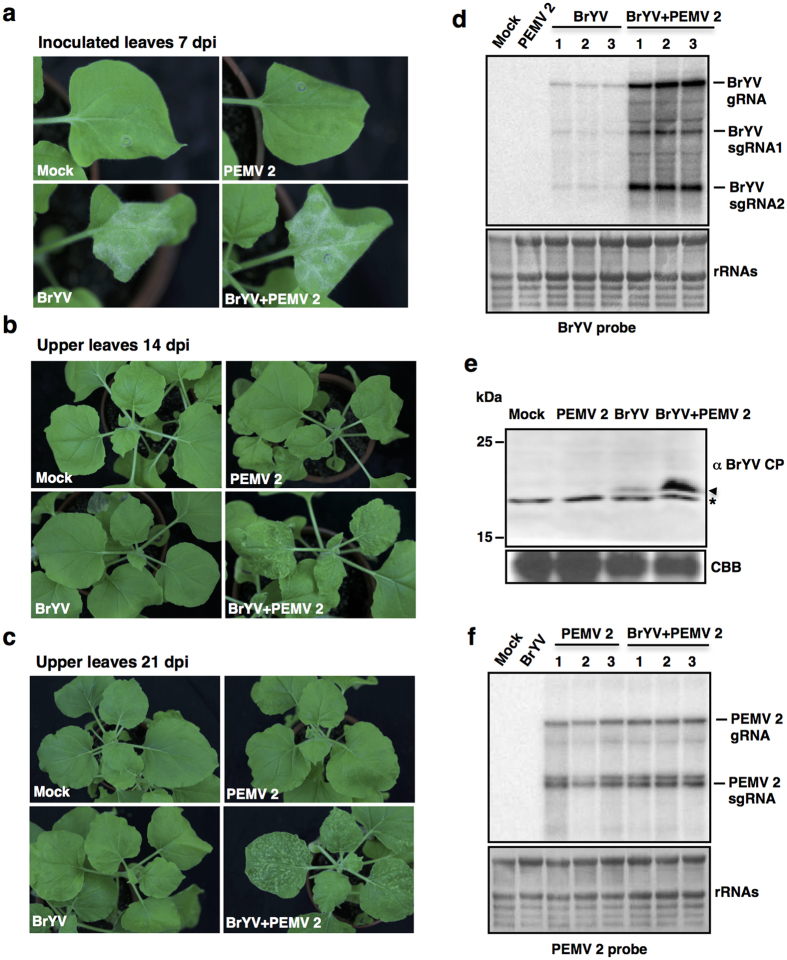
Synergistic infection of Brassica yellows virus (BrYV) and *Pea enation mosaic virus 2* (PEMV 2) in *Nicotiana benthamiana*. (**a**) Agroinfiltration of either BrYV or BrYV + PEMV 2 caused cell death in inoculated leaves by 7 days post inoculation (dpi) at 18 °C. (**b**) and (**c**) Symptoms were induced by co-infection of BrYV + PEMV 2 at 18 °C in upper leaves. Infected plants were photographed at 14 dpi (**b**) and 21 dpi (**c**). (**d**) and (**f**) Northern blot analysis showing accumulation level of BrYV (**d**) and PEMV 2 (**f**) RNAs, respectively, in upper leaves of *N. benthamiana* singly infected with BrYV or PEMV 2, or doubly infected with BrYV and PEMV 2 at 14 dpi. Three independent infected plants were used for detection of BrYV (**d**) and PEMV 2 RNAs (**f**). Positions of the genomic (gRNA) and subgenomic RNA (sgRNA) are indicated on the right. BrYV and PEMV 2 RNAs were detected using ^32^P-labelled DNA probe specific for 5089–5608 nt of BrYV (**d**) and 2797–3202 nt of PEMV 2 (**f**), respectively. Ribosomal RNA (rRNA) bands stained with methylene blue were used as loading control. 5 μg and 3 μg RNA were loaded for detection of BrYV (**d**) and PEMV 2 (**f**), respectively. (**e**) Western blot detection of BrYV CP in systemic leaves at 14 dpi. An antiserum raised against BrYV CP was used for detection. Coomassie brilliant blue (CBB) stained gel was used as loading control. The arrowhead indicates position of BrYV CP and the asterisk shows the non-specific band.

To examine whether synergism occurs between BrYV and PEMV 2, *N. benthamiana* plants at 3–4 leaf stage were agroinfiltrated with empty vector (Mock), BrYV, PEMV 2 and BrYV + PEMV 2, respectively. In inoculated leaves, agroinfiltration of either BrYV or BrYV + PEMV 2 caused cell death by 7 days post inoculation (dpi) at 18 °C (Fig. 1a), while leaves agroinfiltrated with PEMV 2 did not. Upper leaves of plants co-infected with BrYV + PEMV 2 developed mild leaf curling and produced chlorotic spots by 14 dpi (Fig. 1b), most of the chlorotic spots became necrotic by 21 dpi at 18 °C (Fig. 1c), while plants infected with either BrYV or PEMV 2 showed no obvious symptoms in upper leaves (Fig. 1b,c). Total RNA was extracted from upper leaves at 14 dpi for northern blot detection. The accumulation of BrYV genomic and subgenomic RNAs was greatly increased in co-infected plants compared to that in singly infected plants (Fig. 1d). Western blot showed that the expression level of BrYV CP was higher in co-infected plants than in singly infected plants (Fig. 1e). Accumulation of PEMV 2 RNA was not remarkably changed between singly infected and co-infected plants (Fig. 1f). Taken together, synergistic infection of BrYV and PEMV 2 increased the accumulations of both BrYV RNA and CP, while did not have much effect on the accumulation of PEMV 2 RNA.

### BrYV can be mechanically transmitted from plants co-infected with PEMV 2

**Figure 2:**
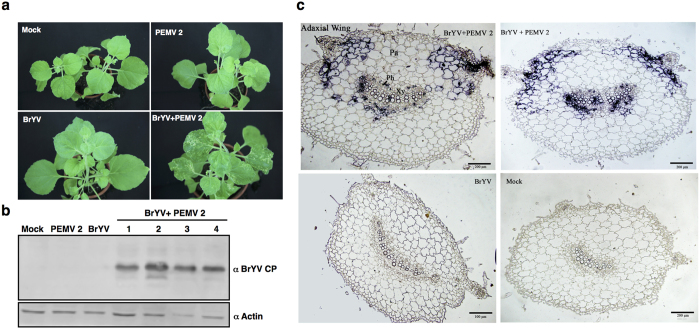
*Pea enation mosaic virus 2* (PEMV 2) helped Brassica yellows virus (BrYV) to be mechanically transmitted and invade non-vascular tissue in *Nicotiana benthamiana*. (**a**) Symptoms were shown in upper leaves of plants mechanically inoculated with extracts containing BrYV and PEMV 2. Water extracts from plants agroinfiltrated with empty vector (Mock), plants infected with BrYV, PEMV 2 or BrYV + PEMV 2, respectively, were used as inoculum for mechanical inoculation. 2–3 leaf stage *N. benthamiana* seedlings were used. Plants were photographed 46 days post inoculation. (**b**) BrYV CP was detected in systemic leaves by western blot when mix infected plants were used as sources for mechanical inoculation. Antiserum raised against BrYV CP was used for detection. Actin protein was used as loading control. (**c**) *In situ* hybridization analysis of BrYV RNA. 3 weeks post inoculation, transverse section of petioles from upper leaves of plants agroinfiltrated with empty vector (Mock), plants singly infected with BrYV or PEMV 2, or doubly infected with BrYV + PEMV 2, respectively, was used for hybridization. Ph, phloem; Xy, xylem; Pa, parenchyma. DIG-labeled antisense RNA from ORF3 of BrYV was used as probe. Blue staining showed the presence of positive-strand RNA of BrYV. Bars = 100 μm or 200 μm.

BrYV belongs to *Polerovirus*, and viruses in this genus cannot be transmitted mechanically^34^. To test whether BrYV can be mechanically transmitted from plants co-infected with PEMV 2, crude extracts of plants that had been infected with BrYV + PEMV 2 were mixed with carborundum and rubbed onto 2–3-leaf stage *N. benthamiana* seedlings. At up to 46 dpi, symptoms appeared only on the plants rubbed with extracts containing BrYV and PEMV 2 (Fig. 2a). Concomitantly, BrYV CP was detected by western blot when co-infected plants were used as sources for inoculation (Fig. 2b). In contrast, extracts from plants infected with BrYV were not infectious via mechanical inoculation (Fig. 2a,b). The results showed that PEMV 2 helped BrYV to be transmitted mechanically.

### BrYV invaded non-vascular tissues when co-infected with PEMV 2

BrYV is a polerovirus and viruses belong to this genus are phloem-restricted^33^,^41^. To examine whether PEMV 2 can help BrYV to invade non-vascular tissues, *in situ* hybridization was performed. Briefly, 3 weeks post inoculation, petioles from upper leaves infected with BrYV, PEMV 2, BrYV + PEMV 2, and mock, respectively, were embedded in paraffin and transverse sectioned to 10 μm thickness and examined for the distribution of BrYV RNA using DIG-labelled antisense RNA from BrYV ORF3. For each treatment, petioles from at least three plants were pooled for *in situ* hybridization. In plants co-infected with BrYV + PEMV 2, viral RNA staining was observed in phloem tissue and parenchyma cells (Fig. 2c). Interestingly, in BrYV and PEMV 2 infected petioles, BrYV signal was observed in just several parenchyma cells near the phloem while considerably greater amount of signal was observed in parenchyma cells surrounding the two adaxial wings with an even pattern (Fig. 2c). The results suggested PEMV 2 can help BrYV to invade non-vascular tissues. However, no signal was observed in petioles of plants singly infected with BrYV (Fig. 2c), likely attributed to the low accumulation level of BrYV in *N. benthamiana* (Fig.1d).

### Profiles of vsiRNAs in *N. benthamiana* plants co-infected with BrYV and PEMV 2

**Figure 3:**
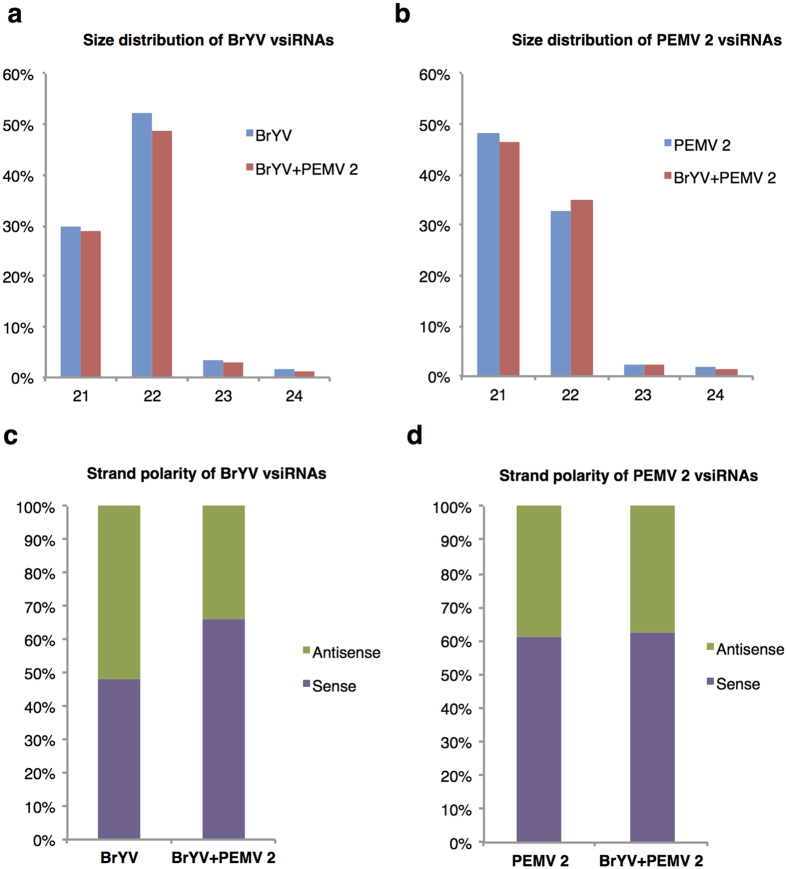
Size distribution and strand polarity of BrYV-derived siRNAs and PEMV 2-derived siRNAs in *Nicotiana benthamiana*. (**a**) Size distribution of BrYV vsiRNAs in size of 21–24 nt in plants infected with BrYV or BrYV + PEMV 2 pair. (**b**) Size distribution of PEMV 2 vsiRNAs in size of 21–24 nt in plants infected with PEMV 2 or BrYV + PEMV 2 pair. (**c**) Strand polarity of BrYV vsiRNAs in BrYV or BrYV + PEMV 2 infected plants. (**d**) Strand polarity of PEMV 2 vsiRNAs in PEMV 2 or BrYV + PEMV 2 infected plants. VsiRNAs, virus-derived siRNAs.

vsiRNAs play pivotal roles in plant defence against viruses^2^,^14^. To characterize the vsiRNAs produced in synergism between BrYV and PEMV 2, small RNA high-throughput sequencing was conducted. Briefly, non-inoculated tissues including leaves and stems from *N. benthamiana* plants inoculated with Mock, BrYV, PEMV 2, and BrYV + PEMV 2 were collected at 14 dpi for RNA extraction and small RNA library construction. The small RNA library preparations were sequenced with IlluminaHiseq 2000 platform and clean reads of 18–40 nucleotides in size were used for further analysis (Table 1). Reads were determined to be vsiRNAs based on 100% sequence identity to the BrYV and PEMV 2 genomes (Table 1) and were mapped for orientation as sense (+) or antisense (−). In plants infected with BrYV, 52,091 reads out of 5,555,111 reads (corresponding to 0.94% of the 18–40 nt reads obtained) were matched to BrYV genome (Table 1), while in BrYV + PEMV 2-infected plants, 24.51% reads were matched to BrYV genome (Table 1). There was a 26-fold increase in the abundance of BrYV-derived siRNAs in co-infected plants compared to that of BrYV infected plants. Percentage of PEMV 2 vsiRNAs in co-infected plants was slightly lower than that of PEMV 2 infected plants (6.07% compared with 9.48%) (Table 1). The results showed that co-infection of BrYV and PEMV 2 substantially increased the accumulation of BrYV vsiRNAs, while slightly decreased the accumulation of PEMV 2 vsiRNAs. Further analysis showed that BrYV vsiRNAs from both BrYV-infected and co-infected plants were predominantly 22 nucleotides in length (52.33% and 48.68%, respectively), followed by 21 nt vsiRNAs (30.03% and 29.07%, respectively) (Fig. 3a). 21 nt size class was the most abundant PEMV 2 vsiRNAs in PEMV 2-infected and co-infected plants (48.24% and 46.59%, respectively) (Fig. 3b). Approximately 30% of PEMV 2 vsiRNAs were 22 nucleotides in length in singly and doubly infected plants (Fig. 3b). Co-infection of BrYV and PEMV 2 did not have obvious effect on the size distribution of both BrYV and PEMV 2 vsiRNAs.

### Co-infection of BrYV and PEMV 2 altered the ratio of positive to negative strand BrYV vsiRNAs

To explore the origin of the vsiRNAs derived from BrYV and PEMV 2, respectively, strand polarity of vsiRNAs was analysed. Almost equal amount of positive and negative-strands BrYV vsiRNAs (48.02% and 51.98% of total BrYV vsiRNAs, respectively) were produced in BrYV-infected plants (Fig. 3c). However, in plants infected with BrYV + PEMV 2, BrYV (+) vsiRNAs accumulated almost 2-fold higher than BrYV (−) vsiRNAs (66.41% compared to 33.59%) (Fig. 3c). For the PEMV 2 vsiRNAs, more abundant (+) vsiRNAs than (−) vsiRNAs were produced in both PEMV 2-infected (61.29% compared to 38.71%) and BrYV + PEMV 2-infected plants (62.82% compared to 37.18%) (Fig. 3d). The results displayed that synergistic infection of BrYV and PEMV 2 increased the percentage of BrYV (+) vsiRNAs, while did not have much effect on strand proportion of PEMV 2 vsiRNAs.

### Mapping vsiRNAs along BrYV and PEMV 2 genomes

**Figure 4:**
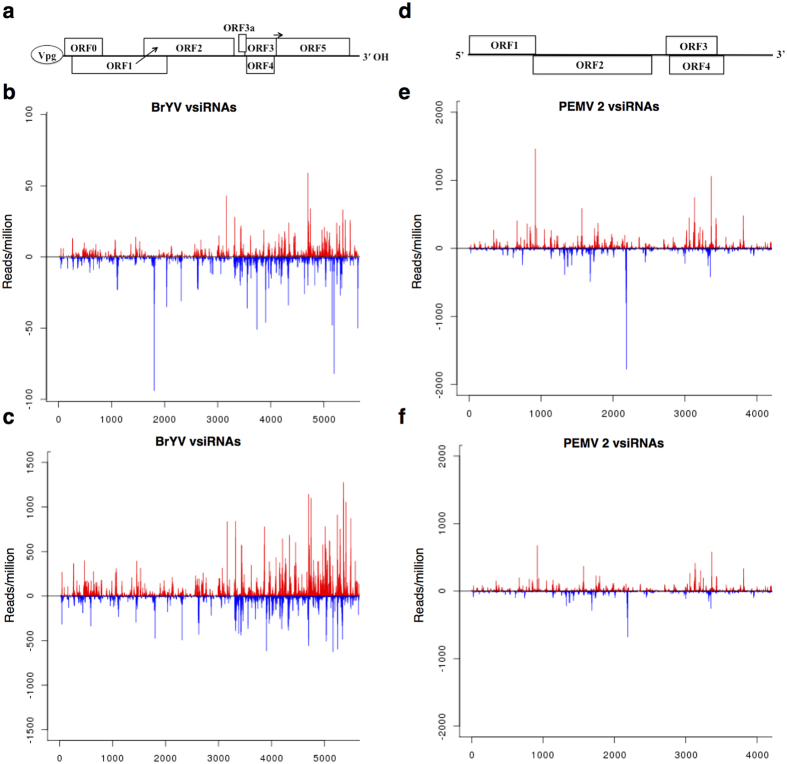
Mapping of perfect match 18- to 32-nucleotide virus-derived small interfering RNAs (vsiRNAs) along the BrYV and PEMV 2 genome. Schematic representation of BrYV (**a**) and PEMV 2 (**d**) genome. Single-base resolution maps of 18- to 32 nt vsiRNAs along the BrYV genome in BrYV (**b**) and BrYV + PEMV 2 (**c**) infected *N. benthamiana* plants. Mapping of 18- to 32 nt vsiRNAs along the PEMV 2 genome in PEMV 2 (**e**) and BrYV + PEMV 2 (**f**) infected plants. Positive- and negative-strand reads are shown in red and blue, respectively. Note that the scale for BrYV vsiRNAs in co-infected plants is 15 times larger than that of BrYV-infected plants.

To further understand the origin of vsiRNAs, 18- to 32 nt vsiRNAs were mapped to the BrYV and PEMV 2 genomes, respectively, at single-base resolution. The results showed that both BrYV vsiRNAs and PEMV 2 vsiRNAs covered almost the entire virus genome in both singly and co-infected plants (Fig. 4). However, there was a dramatic change in the relative amounts and positions to which the BrYV vsiRNAs mapped to the viral genome between BrYV-infected plants and co-infected plants (Fig. 4a–c). Abundance of BrYV vsiRNAs was relatively low in BrYV-infected plants (Fig. 4b). Obvious hotspots can be found in open reading frame (ORF) 2 and ORF5 coding regions in sense strand of BrYV and in ORF2, ORF3/ORF4, ORF5 coding regions and 3′-UTR region in antisense strand (Fig. 4b). In co-infected plants, number of hotspots was increased as co-infection greatly increased the accumulation of BrYV vsiRNAs (Fig. 4c). Most of these hotspots were located at 3′-region of the sense strand of BrYV, especially in ORF5 coding region (Fig. 4c). PEMV 2 vsiRNAs mapped to similar positions in the genome in both singly and doubly infected plants, although the read numbers of vsiRNAs hotspots decreased in co-infected plants (Fig. 4d–f). Most of these PEMV 2 vsiRNAs hotspots occurred within ORFs, notably ORF3/ORF4 and ORF2 (Fig. 4d–f).

### Analysis of the 5′-terminal nucleotide of vsiRNAs

**Figure 5:**
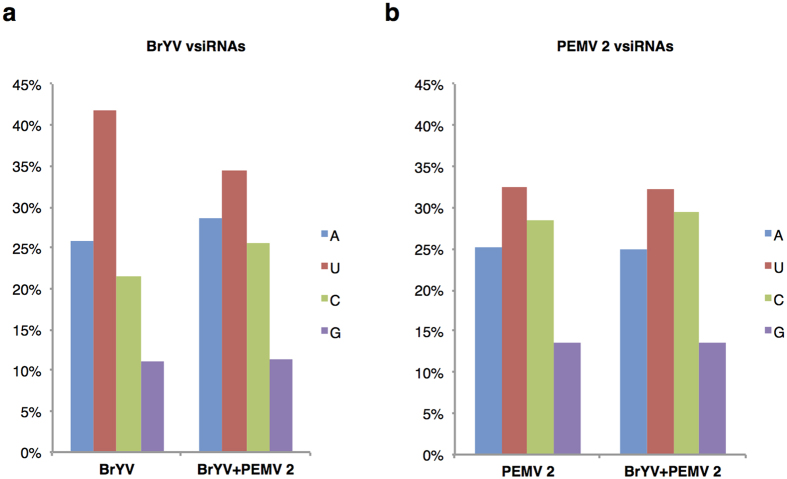
Analysis of the 5′-terminal nucleotide of vsiRNAs. (**a**) 5′-terminal nucleotide of BrYV vsiRNAs in plants infected with BrYV or PEMV 2 + BrYV. (**b**) 5′-terminal nucleotide of PEMV 2 vsiRNAs in plants infected with PEMV 2 or PEMV 2 + BrYV.

Previous studies have shown that sorting of small RNAs into AGO proteins is partially determined by the 5′-terminal nucleotide^42^,^43^. To explore the potential preference of vsiRNAs to AGO proteins during co-infection, 5′-terminal nucleotides of vsiRNAs were analysed. Uracil (U) was the most abundant nucleotide at the 5′-termini of total BrYV vsiRNAs in both BrYV-infected and co-infected plants (Fig. 5a), which were mainly loaded into AGO1, although the percentage was lower in co-infected plants (41.70% compared with 34.55%) (Fig. 5a). Likewise, PEMV 2 vsiRNAs with a U at their 5′ termini were the most abundant and similar percentage was observed in PEMV 2-infected and co-infected plants (32.60% compared with 32.23%) (Fig. 5b). VsiRNAs with 5′-terminal guanine was the least common, accounting for 11.06–13.65% in our dataset (Fig. 5a,b), which is consistent with previous studies^42^,^44^.

### Prediction and annotation of host transcripts targeted by vsiRNAs

To explore the putative plant transcripts targeted by vsiRNAs, the target genes of vsiRNAs were predicted by psRobot, an algorithm to identify targets of plant small RNA^45^. Due to the huge diversity of vsiRNAs, only the top 100 high abundant vsiRNAs derived from plus strand or minus strand were selected for target prediction in *N. benthamiana* plants co-infected with BrYV+PEMV 2 (Supplementary Table S1). Hundreds of target genes were identified and only those with penalty score threshold (0–5, lower is better) not more than 2.0 were listed (Supplementary Table S2). To understand the possible functions of the putative target genes of vsiRNAs, Gene Ontology (GO) analysis using GOseq R package was performed for all predicted target genes of BrYV vsiRNAs and PEMV 2 vsiRNAs. The target genes were annotated into three main categories: biological process, cellular component, and molecular function. As shown in Table 2, for GO terms in biological process, cellular component, and molecular function, only the top 5 groups were listed. GO analysis revealed that for targets of BrYV vsiRNAs, ‘metabolic process’, ‘organic substance metabolic process’, and ‘primary metabolic process’ were the most highly represented groups in term of biological process (Table 2). For the cellular component GO term, ‘nucleus’ was the most highly represented group (Table 2). Under the molecular function GO term, ‘molecular function’ was overrepresented, followed by ‘binding’, ‘catalytic activity’, and ‘ion binding’ (Table 2). With regard to the target genes of PEMV 2 vsiRNAs, in GO terms related to biological process, many target genes were categorized as ‘biological process’, ‘cellular process’, and ‘metabolic process’ (Table 2). Cellular component GO term associated with ‘cellular component’ and ‘membrane’ were overrepresented (Table 2). For molecular function category, ‘molecular function’, ‘binding’ and ‘catalytic activity’ were the top three most abundant groups (Table 2). All other GO terms in each group were presented in Supplementary Table S3.

### Northern blot analysis confirmed that synergistic infection of BrYV and PEMV 2 increased the accumulation of BrYV-derived siRNAs

**Figure 6:**
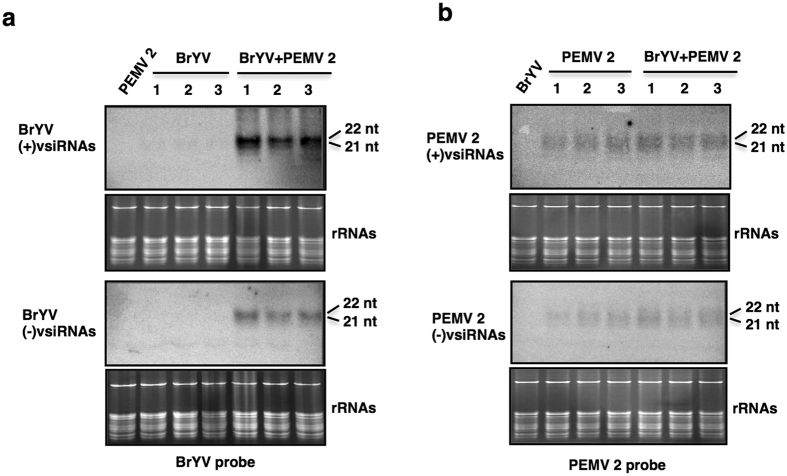
Northern blot analysis of vsiRNAs. Accumulation of Brassica yellows virus (BrYV)-derived small interfering RNA (siRNAs) (**a**) and *Pea enation mosaic virus 2* (PEMV 2)-derived siRNAs (**b**) in upper leaves of plants inoculated with empty vector (Mock), plants singly infected with BrYV or PEMV 2, or doubly infected with BrYV + PEMV 2, respectively. Virus-derived siRNA (vsiRNAs) was detected 14 days after inoculation. 30 μg and 15 μg RNA was loaded for detection of BrYV vsiRNAs (**a**) and PEMV 2 vsiRNAs (**b**), respectively. The vsiRNAs derived from sense and antisense strands of BrYV were detected by ^32^P-labelled DNA oligonucleotides mixture corresponding to BrYV antisense and sense strands (nucleotides 1–40, 241–280, 741–780, 1241–1280, 1741–1780, 2241–2280, 2741–2780, 3241–3280, 3741–3780, 4241–4280, 4741–4780, 5241–5280, 5541–5580), respectively. Similarly, PEMV 2 (+) and (−) vsiRNAs were hybridized by the ^32^P-labelled DNA oligonucleotides mixture corresponding to PEMV 2 (−) and (+) strands (nucleotides 1–40, 241–280, 741–780, 1241–1280, 1741–1780, 2241–2280, 2741–2780, 3241–3280, 3741–3780, 4141–4180), respectively. Three individual infected plants were used for detection of BrYV vsiRNAs (**a**) and PEMV 2 vsiRNAs (**b**). Ribosomal RNA (rRNA) bands stained with ethidium bromide were used as loading control.

To verify the effects of synergistic infection on the biogenesis of vsiRNAs, total RNA was extracted from upper leaves of *N. benthamiana* plants agroinfiltrated with empty vector (Mock), BrYV, PEMV 2 or BrYV + PEMV 2 at 14 dpi, respectively, and northern blot was conducted. The results showed that only very weak signal was detected for BrYV (+) vsiRNAs in BrYV infected plants, whereas in co-infected plants both BrYV (+) and (−) vsiRNAs were readily detected (Fig. 6a). Accumulations of PEMV 2 (+) vsiRNAs and (−) vsiRNAs were not obviously changed in both singly and co-infected plants (Fig. 6b). The results confirmed that co-infection of BrYV and PEMV 2 greatly increased the accumulation of BrYV vsiRNAs while accumulation of PEMV 2 vsiRNAs was not remarkably affected.

## Discussion

Synergistic infections between poleroviruses and umbraviruses are common in nature and can cause important crop diseases^24^,^31^, but the mechanism underlying the interaction is poorly understood. In this study, we showed that co-infection of BrYV and PEMV 2 produced severe symptoms in upper leaves of *N. benthamiana* plants (Fig. 1b,c), which was consistent with previous results^26^,^35^. In addition, accumulations of BrYV RNA and CP increased considerably in systemic leaves of plants infected with BrYV + PEMV 2 compared to that in singly infected plants (Fig. 1d,e), while accumulation of PEMV 2 RNA was not obviously changed (Fig. 1f). Co-infection of BrYV and PEMV 2 appears to be beneficial to BrYV by facilitating the tremendous increase in BrYV RNA and CP, and PEMV 2 is very likely the cause of these synergistic effects. BrYV belongs to *Polerovirus* and viruses in this genus cannot be transmitted mechanically and are restricted to the phloem^32^,^33^,^41^. BrYV was mechanically transmissible from plants co-infected with PEMV 2 (Fig. 2a,b) and invaded non-vascular tissues in co-infected plants (Fig. 2c), in agreement with previous studies^26^,^35^. It has been shown that a *Cucumber mosaic virus* recombinant CMV (ORF4) that express the cell-to-cell movement protein (MP) from the umbravirus *Groundnut rosette virus* (GRV) can complement mechanical transmission of the polerovirus PLRV, while CMVΔ2b (ORF4) in which the virus gene silencing suppressor 2b was untranslatable cannot help PLRV transmission^26^. The results indicated that mechanical transmission of PLRV requires both the cell-to-cell movement function and the ability to defence against RNA silencing^26^.

RNA silencing is crucial in antiviral defence^1^,^2^, which triggers the generation of vsiRNAs during viral infection. It is widely accepted that vsiRNAs play a vital role in the interactions between plants and viruses^46^. Here, we characterized the vsiRNAs from BrYV and PEMV 2 in singly and doubly infected *N. benthamiana* plants to understand the possible roles of vsiRNAs in synergistic infection between poleroviruses and umbraviruses. In plants co-infected with BrYV + PEMV 2, BrYV vsiRNAs showed a 26-fold increase compared to that of plants infected with BrYV (Table 1), which was likely due to the considerably increased accumulation of BrYV RNA during synergistic infection (Fig. 1d), providing greater availability of dsRNA templates for the RNA silencing machinery.

Generation of vsiRNAs from positive-strand plant RNA viruses is dominated by DCL4 and DCL2 in *Arabidopsis thaliana*^2^,^11^. DCL4 is the major protein to process viral dsRNA and generate 21 nt vsiRNAs, while DCL2 acts as a surrogate to produce 22 nt vsiRNAs in the absence of DCL4^11^. Donaire *et al*. (2009) have analysed the vsiRNAs derived from nine viruses belonging to eight different genera. Most of the viruses have 21 nt vsiRNAs as the predominant class, with the exception for tombusvirus *Cymbidium ring spot virus* that accumulates higher level of 22 nt vsiRNAs compared to 21 nt vsiRNAs^44^. In this study, we found that synergistic infection of BrYV and PEMV 2 did not obviously affect the size distribution of both BrYV and PEMV 2 vsiRNAs. In both singly infected and co-infected plants, 22 nt size class was the dominant BrYV vsiRNAs followed by the 21 nt size class (Fig. 3a). Similarly, it has been demonstrated that cotton plants infected with a polerovirus *Cotton leafroll dwarf virus* (CLRDV) has 22 nt vsiRNAs as the predominant class^47^. Maybe it is a unifying feature for genus *Polerovirus* to produce higher level of 22 nt vsiRNAs in plants. The most abundant PEMV 2 vsiRNAs were 21 nucleotides in length in both PEMV 2 infected and BrYV + PEMV 2 infected plants (Fig. 3b), indicating DCL4 is the major Dicer ribonuclease involved in PEMV 2 vsiRNAs biogenesis.

At the beginning, for plant positive-strand RNA viruses, it is assumed that vsiRNAs are originated from the double-stranded replication intermediates^48^. However, Molnar *et al*. show that vsiRNAs are primarily generated from positive-strand viral RNA through sequence analysis of vsiRNAs derived from several positive-strand RNA viruses^49^. Other reports have also suggested that the majority of vsiRNAs are produced from highly structured regions of positive-strand viral RNA rather than the replication intermediates^3^,^44^. In our results, BrYV vsiRNAs were almost equally derived from sense and antisense viral strands in BrYV-infected plants (Fig. 3c), indicating BrYV vsiRNAs were produced from double-stranded replicative intermediates. It is also reported that similar amounts of positive and negative vsiRNAs were observed in cotton plants infected with polerovirus CLRDV^47^. However, in BrYV + PEMV 2 infected plants, PEMV 2 affects the proportions of (+) and (−) strand visRNAs of BrYV, and BrYV vsiRNAs had a strong bias towards the sense strand (Fig. 3c), which suggested BrYV vsiRNAs were mainly derived from highly structures of positive strand during synergistic infection. Positive strand RNA is more abundant than the negative strand during viral infection^50^. In our study, accumulation of BrYV RNA was dramatically increased in BrYV + PEMV 2 infected plants (Fig. 1d), which would make the positive strand of viral RNA more likely to be subjected to the plant RNA silencing machinery, such as DCL proteins or RDRs^18^. It is also possible that virulence factors from PEMV 2 or some unknown host factors contribute to the dominance of sense BrYV vsiRNAs compared to antisense species in co-infected plants. Ratio of positive to negative strand PEMV 2 vsiRNAs was not obviously changed. Percentage of PEMV 2 (+) vsiRNAs was higher than that of PEMV 2 (−) vsiRNAs in plants infected with PEMV 2 or BrYV + PEMV 2 (Fig. 3d), implying PEMV 2 vsiRNAs originated predominantly from highly structured regions of positive strand.

To better understand the origin of BrYV vsiRNAs and PEMV 2 vsiRNAs, single-base resolution maps of 18- to 32 nt vsiRNAs along the BrYV and PEMV 2 genomes, respectively, were created. The relative abundance and distribution of BrYV vsiRNAs along the viral genome varied considerably between BrYV-infected plants and co-infected plants. BrYV vsiRNAs accumulated at a relatively low level in BrYV-infected plants (Fig. 4b). Obvious hotspots were observed in ORF2 coding region and 3′ region of both the positive and negative strand viral RNA (Fig. 4b). It has been reported that polerovirus PLRV contains three subgenomic RNAs (sgRNAs) at 3′-region of viral RNA^51^,^52^. Translation of sgRNA1 enables expression of ORFs 3a, 3, 4, and 5^41^,^53^, while that of sgRNA2 codes two proteins at the 3′-region of ORF5^51^. The recently identified sgRNA3 encodes a RNA-binding protein^52^. Our northern blot result implied that BrYV contained two sgRNAs (Fig. 1d), although the generation of sgRNA3 has not been described. It seems that vsiRNAs hotspots identified at the 3′ region might be own to expression of these sgRNAs, making the 3′ region more accessible by the RNA silencing machinery. In plants co-infected with BrYV + PEMV 2, hotspots were mostly identified at 3′-region of the positive strand (Fig. 4c), in according with our hypothesis that BrYV vsiRNAs were produced from hairpin regions of positive strand during co-infection. Remarkably high vsiRNAs densities were located in ORF5 of sense strand in BrYV + PEMV 2 infected plants (Fig. 4c), which might be due to the relatively high accumulation of sgRNA2 in co-infected plants (Fig. 1d).

Taken together, we showed that co-infection of BrYV and PEMV 2 on *N. benthamiana* led to severe symptoms and dramatically increased the accumulation of BrYV. Tissue tropism of the phloem-restricted BrYV was also altered in presence of PEMV 2. We also characterized the vsiRNAs derived from BrYV and PEMV 2 in singly and doubly infected plants by small RNA deep sequencing to understand the possible effects of synergism have on vsiRNAs from BrYV and PEMV 2. Future studies should focus on unravelling components of PEMV 2 and the host factors and/or microRNAs that might be involved in synergism between BrYV and PEMV 2.

## Methods

### Plant material

All *N. benthamiana* plants were grown and maintained at 18 °C with 16 h light and 8 h dark.

### Modification of PEMV 2 infectious clone

Full-length cDNA infectious clones of BrYV were constructed as described^54^. In this study, infectious clone BrYV-5B3A was used. Plasmid pPEMV 2 containing the full-length cDNA of PEMV 2 under control of T7 RNA polymerase promoter was kindly provided by Dr. Michael Taliansky (The James Hutton Institute, Scotland)^26^. To construct a PEMV 2 infectious cDNA clone under control of a duplicated *Cauliflower mosaic virus* 35 S promoter, full-length sequence of PEMV 2 was amplified from plasmid pPEMV 2 using primer pair PEM2-001F/PEM2-Bg4253R (Supplementary Table S4). The resulting products were then digested with *Bgl*II and ligated between the *Stu*I and *Bam*HI sites of vector pCass4-Rz^55^. The new infectious clone was named pCaPE2.

### Agrobacterium-mediated inoculation

The *Agrobacterium tumefaciens* GV3101 provided by Dr. Baulcombe was transformed with each plasmid through a freeze-thaw method as described^56^. Agrobacterium was grown in Luria-Bertani broth containing kanamycin and tetracycline (100 μg/mL each) with shaking (200 rpm) at 28 °C. After reaching OD_600_ = ∼0.5, Agrobacterium cultures were harvested by centrifugation at 4000 rpm for 10 min. Finally, Agrobacterium pellets were resuspended in infiltration buffer (10 mM MES, 10 mM MgCl_2_, and 150 μM acetosyringone) and incubated for at least 3 h at room temperature before inoculation. For single inoculation of BrYV or PEMV 2, *A. tumefaciens* cultures (OD_600_ = 0.5) containing the relevant constructs were used. For co-inoculation of BrYV and PEMV 2, the same amount of *A. tumefaciens* cultures (OD_600_ = 0.5) for each virus was used. 2 ml syringe without needle was used for infiltration.

### Western blot

Total protein extraction and western blotting were performed as described^57^. Antiserum raise against BrYV CP were used to detect expression of BrYV CP in *N. benthamiana*.

### RNA extraction and RNA blot analysis

Total RNA from leaves of *N. benthamiana* was extracted with Trizol reagent (Invitrogen, USA). Detection of high-molecular-weight RNA and small RNAs was conducted as described^57^. Briefly, for detection of high-molecular-weight RNA, 5 μg and 3 μg of total RNA was separated on 1% denaturing agarose gel for detection of BrYV and PEMV 2 RNAs, respectively, and transferred to Hybond-N^+^ membranes. [α-^32^P] dCTP-labelled DNA probes specific for nt 5089–5608 of BrYV, or nt 2797–3202 of PEMV 2 were used for hybridization. 30 μg and 15 μg RNA was loaded for detection of BrYV vsiRNAs and PEMV 2 vsiRNAs, respectively. The vsiRNAs derived from (+) and (−) strand of BrYV were detected by the ^32^P-labelled DNA oligonucleotides corresponding to minus and plus strand of BrYV (nucleotides 1–40, 241–280, 741–780, 1241–1280, 1741–1780, 2241–2280, 2741–2780, 3241–3280, 3741–3780, 4241–4280, 4741–4780, 5241–5280, 5541–5580) (Supplementary Table S4), respectively. Similarly, vsiRNAs derived from (+) and (−) strand of PEMV 2 were hybridized by the ^32^P-labelled DNA oligonucleotides corresponding to (−) and (+) strand PEMV 2 (nucleotides 1–40, 241–280, 741–780, 1241–1280, 1741–1780, 2241–2280, 2741–2780, 3241–3280, 3741–3780, 4141–4180) (Supplementary Table S4), respectively.

### Mechanical inoculation

Water extracts (for 0.1 g leaves, 500 μl ddH_2_O was added) from plants agroinfiltrated with empty vector (Mock), plants infected with BrYV, PEMV 2 or BrYV + PEMV 2, respectively, were mixed with carborundum and used as inoculum for mechanical inoculation. 2–3 leaf stage *N. benthamiana* seedlings were used.

### *In situ* hybridization

Tissue fixation and *in situ* hybridization were performed as described^58^. Briefly, 3 weeks post inoculation, petioles from upper leaves were fixed in 3.7% formalin/acetic acid/alcohol (FAA) solution at 4 °C overnight and then dehydrated with graded ethanol solutions. Next, samples were embedded in paraffin and transverse sectioned to 10 μm thickness and assayed for the distribution of BrYV RNA using DIG-labelled BrYV ORF3 antisense RNA probe. After hybridization, samples were washed and incubated with alkaline phosphatase-conjugated DIG antibody (Roche). Finally, signal was visualized with NBT/BCIP solution.

For RNA probe preparation, ORF3 of BrYV was amplified from plasmid BrYV-5B3A using primer pair BrA-P3-EcoRF/BrA-P3-HindR (Supplementary Table S4). The resulting fragment was inserted into the restriction sites between *Eco*RI/*Hin*dIII of pSPT19 (Roche Applied Science, Germany) to give pSPT19-BrORF3. DIG-UTP-labelled antisense RNA probe of BrYV was generated by DIG RNA labeling kit using T7 RNA polymerase (Roche Applied Science, Germany), with pSPT19-BrORF3 as template.

### Sample preparation, small RNA sequencing, and bioinformatics analyses

Samples were collected from non-inoculated tissues including leaves and stems of mock-inoculated plants or plants infected with BrYV, PEMV 2 and BrYV + PEMV 2, respectively, at 14 dpi under 18 °C. Total RNA was extracted with Trizol reagent (Invitrogen, USA) following the manufacturer’s instructions. The concentration and quality of total RNA was determined by a spectrophotometer (Nanodrip ND-2000, ThermoFisher Scientific, USA) and agarose gel electrophoresis (1%). Small RNA libraries were constructed using IlluminaTruSeq^TM^ Small RNA Sample Preparation Kit (Illumina, San Diego, USA) according to manufacturer’s instructions and index codes were added to attribute sequences to each sample. The resulted cDNA was then amplified using a common primer and a primer containing one of the 48 index sequences. DNA fragments with adapter on both ends were enriched using Illumina PCR Primer Cocktail in a 12 cycles PCR reaction. Products were purified and quantified using the Agilent high sensitivity DNA assay on the Agilent Bioanalyzer 2100 system. The clustering of the index-coded samples was performed on a cBot Cluster Generation System using TruSeqSE Cluster Kit (Illumina) following the manufacturer’s instructions. After cluster generation, the library preparations were sequenced on an IlluminaHiseq 2000 platform and 50 bp single-end reads were generated.

The low quality reads and adaptor sequences from raw reads were removed by Perl and Python scripts to obtain the clean reads. Small RNAs ranging from 18 to 40 nucleotides in size from clean reads were extracted and mapped to the BrYV or PEMV 2 genomes by Bowtie^59^. Only the small RNAs showed perfect matches to the sense or antisense viral genomic sequences were identified as vsiRNAs.

### Prediction and annotation of vsiRNAs target genes

The psRobot program was used to predict plant transcripts targeted by vsiRNAs generated from BrYV and PEMV 2 in co-infected *N. benthamiana* plants^45^. The GO analysis was conducted to annotate the predicted target genes by GOseq R package. The target genes were categorized into three categories: biological process, cellular component, and molecular function.

## Additional Information

**How to cite this article:** Zhou, C.-J. *et al*. Synergistic infection of BrYV and PEMV 2 increases the accumulations of both BrYV and BrYV-derived siRNAs in *Nicotiana benthamiana. Sci. Rep.* **7**, 45132; doi: 10.1038/srep45132 (2017).

**Publisher's note:** Springer Nature remains neutral with regard to jurisdictional claims in published maps and institutional affiliations.

## Supplementary Material

Supplementary Information

## Figures and Tables

**Table 1:** Classification and abundance of siRNAs mapping to BrYV and PEMV 2 genomes in each library.

Category	Reads obtained from each library			
	Mock	BrYV	PEMV 2	BrYV + PEMV 2
Total raw reads	8,579,560	9,600,671	8,489,083	10,347,694
Total clean reads	8,226,079	8,960,544	8,168,039	10,130,560
Clean reads (18–40 nt)	5,290,127	5,555,111	5,923,826	8,749,693
BrYV-derived siRNAs (100% match)	—	52,091	—	2,144,771
PEMV 2-derived siRNAs (100% match)	—	—	561,298	531,340

**Table 2:** Top 5 GO terms in biological process, molecular function, and cellular component of the predicted target genes of vsiRNAs.

Term type	GO accession	Description	Number of genes
**BrYV**			
Biological process	GO:0008152	Metabolic process	166
Biological process	GO:0071704	Organic substance metabolic process	141
Biological process	GO:0044238	Primary metabolic process	136
Biological process	GO:0044699	Single-organism process	85
Biological process	GO:0044763	Single-organism cellular process	81
Cellular component	GO:0005634	Nucleus	24
Cellular component	GO:0044422	Organelle part	17
Cellular component	GO:0044446	Intracellular organelle part	17
Molecular function	GO:0003674	Molecular function	274
Molecular function	GO:0005488	Binding	190
Molecular function	GO:0003824	Catalytic activity	175
Molecular function	GO:0043167	Ion binding	121
Molecular function	GO:1901363	Heterocyclic compound binding	113
**PEMV 2**			
Biological process	GO:0008150	Biological process	234
Biological process	GO:0009987	Cellular process	187
Biological process	GO:0008152	Metabolic process	181
Biological process	GO:0071704	Organic substance metabolic process	147
Biological process	GO:0044237	Cellular metabolic process	145
Cellular component	GO:0005575	Cellular component	148
Cellular component	GO:0016020	Membrane	81
Cellular component	GO:0044425	Membrane part	46
Cellular component	GO:0043231	Intracellular membrane-bounded organelle	35
Cellular component	GO:0031224	Intrinsic to membrane	29
Molecular function	GO:0003674	Molecular function	295
Molecular function	GO:0005488	Binding	194
Molecular function	GO:0003824	Catalytic activity	179
Molecular function	GO:0097159	Organic cyclic compound binding	103
Molecular function	GO:1901363	Heterocyclic compound binding	103
