# Supplementary material for: Synergistic infection of BrYV and PEMV 2 increases the accumulations of both BrYV and BrYV-derived siRNAs in Nicotiana benthamiana
Source: Sci Rep. 2017 Mar 27;7:45132. doi: 10.1038/srep45132 (PMC5366869; doi:10.1038/srep45132)
Supplement: Supplementary Information [file srep45132-s1.pdf]

Synergistic infection of BrYV and PEMV 2 increases the accumulations of both BrYV and BrYV-derived siRNAs in *Nicotiana benthamiana*

CUI-JI ZHOU, XIAO-YAN ZHANG, SONG-YU LIU, YING WANG, DA-WEI LI, JIA-LIN YU AND CHENG-GUI HAN*

State Key Laboratory for Agro-Biotechnology and Ministry of Agriculture Key Laboratory for Plant Pathology, China Agricultural University, Beijing, 100193, P.R. China

Supplementary Table S1 Top 100 vsiRNAs from BrYV and PEMV 2 selected for target prediction. Numbers of position indicate vsiRNAs starting positions of the sense (+) strand or ending positions of the antisense (-) strand of viral genomes.

BrYV vsiRNAs				PEMV 2 vsiRNAs			
Sequence	Position	Strand	Counts	Sequence	Position	Strand	Counts
AAGAGACUUAAGUAAAACCACU	3322	+	7007	UUUGGUAGGGGCUGUGCUGCC	922	+	5564
UGUUUCUCAGUUCUGAACGAGU	3164	+	6960	AGCCACCGGUAGAACGACGUGU	2166	-	4291
CUCAACUGUGCUCGUUGGUAGU	5493	+	6484	AUGUCGAACAGGUAUGCCAUG	3436	+	1631
UUCUUUGGAUGAGAAAGACGGA	4746	+	6345	CUGGAGAAAAUAGAGUUCUGC	1985	+	632
UACGAACAGGACCUAGCUGAGU	5296	+	6252	ACUGUGUCGCUAGAAUCAAGC	25	+	615

CACACUGUAAACUCAAUUCACU	3866	+	5922	AAGACUGAAACUCAAGCUGAGU	3132	+	580
UUCCAGUCCCGGAAGGAACGUG	4337	+	5382	UUUCCGUAGAGAAAGUGGUAGU	3017	+	542
AGAGGAGCUGAUCCAGAACGGU	2344	+	5057	CUGUCGUCGUGGGACUAGACG	1569	+	527
UUUCUUUGGAUGAGAAAGACGG	4745	+	4869	UCGGCUGGGGGAAGACACUAG	943	+	511
UUUGUUAAUCGUUGAGGAGAGU	3885	-	4842	ACAAUGCUAGGACAGAACCCC	1140	+	397
CGUGAUGAUGGUGAACUCAUG	5359	+	4778	UAGGACCGGUUGUGCUUCUUCC	4194	+	395
AGGGCUUUAUGGGAAGAUUUGU	5138	-	4734	UGACAAUAAAAGAACUCGAGG	2858	+	331
AGAGACUUAAGUAAAACCACU	3323	+	4685	UUUGGCUCUGAGCUCCAUGGU	720	-	323
ACCUAAGAAUCUCUAAACACGU	3436	+	4672	UGGAUGUCUCGCCCAAAAGAUC	3928	+	323
CGAUUCCAUUUAUCUGUAUGAU	4206	+	4452	CUGUCGUCGUGGGACUAGACGA	1569	+	294
UCAGAGAUAGAGGAAUUGUAGU	5030	+	4060	CUCCUGCGGGAUAACUGUGUC	801	+	291
ACGAACAGGACCUAGCUGAGU	5297	+	4008	UGGAGAAGUUCAACAAUGCUG	1905	+	273
AUCUCCCAGCUGUAGAGCGAGG	1785	-	3981	UAUGAUCUUUUGGGCGAGACAU	3931	-	273
CGUGAUGAUGGUGAACUCAUGG	5359	+	3981	AAGGGAGCGAUGUCGAACAGG	3427	+	272
CUGAGAAGAUCGUCAUUCUGAG	5359	+	3980	AGCCCUCAGGAUAGUGAGCUCU	3764	+	268
CAGAGAUAGAGGAAUUGUAGU	5031	+	3974	UUGUCCGUGUAGAACACCCUU	1123	-	267
AUCAAUCAGAAGUGUGGAACGU	4454	+	3913	AAGGUCAAAGGUAGACGCAUG	1745	+	251
UCUUCUGUAGUCGGAUGAAGGU	2291	-	3778	CAUACUGAUCUCUAUAAAU	5	-	249
UGGACAGAUCGUGAUAUGGAGU	4708	+	3647	CACCGAGCUGUUCGAAAGCAAU	3243	+	247
ACCCACACUGUAAACUCAAUU	3863	+	3618	AAGAGUGGGACCAAGUGCCAGU	2201	+	244
UUAACGAUUUCGCGUACGUGU	3452	-	3530	UUGUGGGUCACUGCUUCGAUG	3315	-	242
ACUGAUCACGGAUAUUGGUAUC	3401	-	3312	CCAAUUUGCAGAACGAUGGU	3364	+	228
AUUAACGAUUUCGCGUACGUGU	3452	-	3223	CCAGGACAAUGUUGAAUCGUG	1410	-	225
CUUUUCAAACUUCUGUGGUACU	5315	-	3192	AAGACUGAAACUCAAGCUGAA	3132	+	217
AGACCUCGUCGAGAAGGACACU	4789	+	3182	AUGUCGAACAGGUAUGCCAUA	3436	+	213
UGAGGCUCAGAAUGACGAUCUU	5007	+	3171	ACUGUGUGCUCAUGGUGUUGC	1794	+	210
UCAACUGUGCUCGUUGGUAGUG	3763	+	3168	AGCAUUGUUGAACUUCUCCAAG	1903	-	194
UCGUUCUCUGCUCUAUCAGCUU	199	+	3146	AGUGGUCUGGCUGUGUGUGGU	2132	+	182
UGAAACGGCGUAGUUGUACUUC	4677	-	3146	CCGAGCUGUUCGAAAGCAACU	3245	+	180
AACCGGCUUCAGUUUUCUGAGA	5223	-	3142	CUAGCCUGUGGUCGGCGAGC	894	+	180

CGAUUUCUGUCAAUCUGGACU	473	+	3086	CAGUACGGAGCUGACCCUGCU	2959	-	176
UUCAGACGUUGUAGUUCCAACU	2605	-	3021	UGACAUGGACACCUCUUUGGG	1771	+	176
UCCUUGUUGGAAAUACCUGACU	5059	+	3020	UCAAAGGUAGACGCAUGAGCGG	1749	+	175
GAUACUGAUCACGGAUAUUGGU	3404	-	2925	CAUUGUCCGUGUAGAACACC	1126	-	174
UUACUGUUGCAGAAAACUCUUU	3302	-	2876	CAAGUAAUUGUGGGUCACUGU	3322	-	172
CGCGUAGAAGCUCGCCAAGAUU	569	-	2810	UGGAAUGCCAUCGCCCAGAGUG	2114	+	169
UCCAGUACGAACAGGACCUAGC	5291	+	2804	CGAUGCGUGGACUCCACAUGA	3299	-	161
AAACAUGAACUGGACCAACGUG	4263	+	2793	UUAGCAACUGCAGGGUACUUGU	1485	-	156
CAAAGACAAGACUCUAAAACU	4882	+	2772	UGCGUCCUCUUGGAGAUUUUG	1056	+	156
UUGAUGAAAAACGCUGGAGUC	5404	+	2725	CAAUUUGCAGAACGAUGGUAC	3365	+	153
AACCUGCUAGGAAUUUGUAAUC	3357	-	2680	AUGGACACCUCUUUGGGCAAC	1775	+	153
AUGAAACGGCGUAGUUGUACUU	4678	-	2646	ACAUGAGAAACAGGCUGAAUG	3284	-	149
UAAGAAUCUCUAAACACGUACG	3439	+	2632	UGGAGCCGAUGCUGUACAAAGU	1449	+	149
CGGGACUCGAAUGUUUCUUCC	4965	+	2601	CAAUUUGCAGAACGAUGGUGG	3365	+	145
UACUGUUGCAGAAAACUCUUU	3302	-	2573	CAGCCGCAUGGAUAACUCCUGG	691	+	144
CAUGAUUGCCUAUUCUGAUGAU	4434	+	2564	UUACAGUAGGAGGGGACAGUGC	3671	+	142
CUGGCUGAUUUCGCUGAAUGCG	3535	-	2540	CCGUGACUUGAUGAACCCUCU	1649	-	141
ACAACCGGACAGGAAGAACUGU	3617	+	2400	GGCAACUGUGUGCUCAUGGUGC	1790	+	140
GACACAGAUCCUUGGGCUGAGG	5107	+	2367	UCGACGUUGUGCGUCCUCUUGA	1047	+	140
UACUCAACUGUGCUCGUUGGU	5491	+	2357	CUCAAGUAAUUGUGGGUCACUG	3323	-	139
GUUGAUGAAAAACGCUGGAGUC	5403	+	2344	GCCCUCAGGAUAGUGAGCUCU	3765	+	138
UUCUUUGGAUGAGAAAGACGG	4746	+	2331	AAUUUGGCUCUGAGCUCCAUGG	721	-	135
UCGAUUUCAAAUCUAAGACUGG	1456	+	2287	GGGCAUCUUUAGGACUCAGU	1306	-	133
UCCACGGAUGAGCAAGUGCUGG	2558	+	2285	ACGACAGGAUCUUUGAAACU	1556	-	133
UCAAUCAGAAGUGUGGAACGUU	4455	+	2262	GCAUCUUUAGGACUCAGUGGGU	1302	-	130
GAUAGUUUAUACUGGCGUCUGU	4155	+	2203	GACAAUGCUAGGACAGAACCU	1139	+	129
CCUAAGAAUCUCUAAACACGU	3437	+	2189	AGCGAUGUCGAACAGGUAUGA	3432	+	128
CACAGAUCCUUGGGCUGAGGU	5109	+	2189	AUGCCCGGGUCAAGACGUUUG	1320	+	127
AUUGAGAAUGAAAACAUGAACU	4252	+	2187	CUGGGACCCAGUGUAACUCUCU	1240	-	126
UAAAAGACUAGGAUUCAAGGU	2989	+	2175	UGAGACGGGGGAGAUCAUUGC	1522	+	125

UGAUGGUGAACUCAUGGAAAUG	5364	+	2162	UCACCUUUUCGCGUCGACUGU	2706	+	123
CAAUGAAUUGCAUCAACUUAGG	23	-	2151	GCGGAUGGGUAAUAUAUGACG	2766	+	121
UACUUGUUCCUGAACCAGAGCU	267	+	2141	GUUUGGCAGAACUCUAUUUUC	1990	-	118
GACAGAUCGUGAUAUGGAGUU	4710	+	2139	UAACGCCUCAACAGACACAUG	1607	-	118
CUUCAGACGUUGUAGUUCCAAC	2606	-	2085	UCCUGCGGGAUAACUGUGUC	802	+	114
GAUGGAUAGUUUAUACUGGCGU	4151	+	2075	UAGGUCUGGACCUUUUCUCUA	598	-	113
UCCCGUUGAUGAAAGACGCUGU	3940	-	2035	GCCGAUAUACACGUCGUUCU	2158	+	112
UGUGCUCGUUGGUAGUGAUACG	5499	+	2028	AAGACUGAAACUCAAGCUGAAC	3132	+	108
UGGCUGAUUUCGCUGAAUGCG	3535	-	2023	GUGGUCUGGCUGUGUGUGGCG	2133	+	108
UGACGUUAUUACUUGAAUACC	4295	-	2018	UAGGACCGGUUGUGCUUCUUC	4194	+	107
CGGACGAUUCCAUUUAUCUGU	4202	+	2003	GGUUGAACCGCCGGUUGACGU	1963	+	106
GAGAAGGACACUCGAAGGCAGU	4799	+	1998	CUGUGUGCUCAUGGUGUUGCUU	1795	+	106
AUUUCGCGUACGUGUUUAGAGA	3445	-	1989	GAUAACUCCUGGCUGCAUUGC	701	+	105
UGUAACCGGCUUCAGUUUUCUG	5226	-	1976	GGGGGACUGCUUUCCGUAGAG	3007	+	105
AAACAGUCGAAGUACAACUACG	4669	+	1970	CAUACUGAUCUCUAUAAAUACCC	1	-	104
ACCAGUCAAAGACAAGACUCU	4876	+	1970	AAAUGGUUCUGUGGGUGCCUCU	424	+	104
CUAUCCAUCUGUACUCUUUAGU	4138	-	1968	UUGCUGUUGGGAUGCCACCUC	3400	-	103
ACAGAUCGUGAUAUGGAGUUG	4711	+	1941	AAUACCUGAAGGGAGCGAUGU	3419	+	103
GAUAGAGGAAUUGUAGUAGACC	5035	+	1940	AUCUUUAGGACUCAGUGGGUU	1301	-	100
UGAUGAAAGACGCUGUAAACGU	3934	-	1937	UGAACACUACUACCACUUUCU	3026	-	100
AGGACUGUUAGGCUGCAAGGC	1073	+	1934	CAACUGUGUGCUCAUGGUGCU	1792	+	100
AUGAACUGGACCAACGUGGACG	4267	+	1918	AAUAGAGUUCUGCCAAACGCA	1993	+	99
UCAGGGACAGGUUCUAGAGAUG	4572	+	1912	CCAGGACAAUGUUGAAUCGUGG	1409	-	99
UUGACGUUAUUACUUGAAUACC	4295	-	1900	CCAAGACUGAAACUCAAGC	3130	+	99
UCUGUAGAACUCUGAUCAAAGC	4172	+	1875	UCAUCCGUUAUUAGCUGGGACU	2078	+	98
UCUGAGAAGAUCGUCAUUCUG	5014	-	1857	UGCAAAUUGGCAGAGCACGUG	3353	-	95
UAACUUCAGACGUUGUAGUUCC	2609	-	1856	UUGUCCUGGCUAAAUACAUCA	1422	+	94
UUGUUUGGGCUUUGAUCAGAGU	4180	-	1856	CGAGCUUUUCAACAAUGGUGA	1852	+	93
ACAUGAUCGUCCGCAAUAGACU	2712	+	1854	UCAAGUAAUUGUGGGUCACUGU	3322	-	91
UUAAGUCUCUUACUGUUGCAGA	3311	-	1854	CAAAGAGACAUCACUGUAGGC	2527	-	91

UGAACUGGACCAACGUGGACG	4268	+	1853	CCUCAGGAUAGUGAGCUCC	3767	+	89
AUACUUGUUCCUGAACCAGAGC	266	+	1837	CAUGGAUAACUCCUGGCUGCA	697	+	89
UCUUUGGAUGAGAAAGACGGAU	4747	+	1831	AUGGAUAGGGUUGUGGAGAGUU	1226	+	89
CUUUGGAAUAGAGCUUUUCU	1424	-	1802	UAAUUGUGGGUCACUGCUUCG	3318	-	87
UUCAAACUUCUGUGGUACUCAGC	5311	-	1797	CUGCCAAUUUGCAGAACGAUG	3361	+	86

Supplementary Tabe S2 Predicted target genes of the selected vsiRNAs

Sequence	Target ID	Position	Penalty score threshold (0-5, lower is better)
**BrYV vsiRNAs**			
AAGAGACUUAAGUAAAACCACU	Niben101Scf07589g02005	1266	2.0
UUCUUUGGAUGAGAAAGACGGA	Niben101Scf04627g02017	595	1.2
UACGAACAGGACCUAGCUGAGU	Niben101Scf10384g01007	1275	2.0
UUUCUUUGGAUGAGAAAGACGG	Niben101Scf04627g02017	596	1.5
UUUGUUAAUCGUUGAGGAGAGU	Niben101Scf04556g04014	672	2.0
UUUGUUAAUCGUUGAGGAGAGU	Niben101Scf06342g03003	2064	1.2
UUUGUUAAUCGUUGAGGAGAGU	Niben101Scf07199g01007	1378	1.2
CGUGAUGAUGGUGAACUCAUG	Niben101Scf00712g06009	1342	2.0
CGUGAUGAUGGUGAACUCAUG	Niben101Scf01369g05021	1284	2.0
AGAGACUUAAGUAAAACCACU	Niben101Scf07589g02005	1265	2.0
CGAUUCCAUUUAUCUGUAUGAU	Niben101Scf04183g00005	845	2.0
UCAGAGAUAGAGGAAUUGUAGU	Niben101Scf08419g01038	370	2.0
ACGAACAGGACCUAGCUGAGU	Niben101Scf10384g01007	1274	2.0
CAGAGAUAGAGGAAUUGUAGU	Niben101Scf01497g05008	693	2.0
CAGAGAUAGAGGAAUUGUAGU	Niben101Scf02772g03006	338	1.8
CAGAGAUAGAGGAAUUGUAGU	Niben101Scf04094g00001	100	2.0
CAGAGAUAGAGGAAUUGUAGU	Niben101Scf08419g01038	369	2.0
AUCAAUCAGAAGUGUGGAACGU	Niben101Scf06158g00003	354	1.8

AUCAAUCAGAAGUGUGGAACGU	Niben101Scf20553g00005	1604	2.0
UUAACGAUUUCGCGUACGUGU	Niben101Scf03526g01023	365	2.0
AUUAACGAUUUCGCGUACGUGU	Niben101Scf03526g01023	366	2.0
CUUUUCAAACUUCUGUGGUACU	Niben101Scf13227g00027	1182	1.5
CUUUUCAAACUUCUGUGGUACU	Niben101Scf15965g00015	1286	1.5
AGACCUCGUCGAGAAGGACACU	Niben101Scf05367g00005	169	2.0
UGAGGCUCAGAAUGACGAUCUU	Niben101Scf17663g00002	143	2.0
UCGUUCUCUGCUCUAUCAGCUU	Niben101Scf03885t08009	34	2.0
UCGUUCUCUGCUCUAUCAGCUU	Niben101Scf03929t01012	34	2.0
UCGUUCUCUGCUCUAUCAGCUU	Niben101Scf11531t00007	34	2.0
UGAAACGGCGUAGUUGUACUUC	Niben101Scf06795g03005	552	2.0
CGAUUUCUGUCAAUCUGGACU	Niben101Scf00369g11013	1532	2.0
CGAUUUCUGUCAAUCUGGACU	Niben101Scf03937g02019	1176	2.0
AAACAUGAACUGGACCAACGUG	Niben101Scf01453g02010	4177	2.0
CAAAGACAAGACUCUAAAACU	Niben101Scf13185g02001	1119	2.0
UUGAUGAAAAACGCUGGAGUC	Niben101Scf00463g00019	4981	2.0
AUGAAACGGCGUAGUUGUACUU	Niben101Scf06795g03005	553	2.0
UACUGUUGCAGAAAACUCUUU	Niben101Scf05294g00030	654	1.5
CAUGAUUGCCUAUUCUGAUGAU	Niben101Scf00558g00005	3837	2.0
CAUGAUUGCCUAUUCUGAUGAU	Niben101Scf00825g04001	1678	2.0
CAUGAUUGCCUAUUCUGAUGAU	Niben101Scf01785g01010	1249	2.0
CUGGCUGAUUUCGCUGAAUGCG	Niben101Scf03504g03006	385	2.0
CUGGCUGAUUUCGCUGAAUGCG	Niben101Scf05880g01001	951	2.0
UUCUUUGGAUGAGAAAGACGG	Niben101Scf04627g02017	595	1.2
UCAAUCAGAAGUGUGGAACGUU	Niben101Scf06158g00003	353	1.8
AUUGAGAAUGAAAACAUGAACU	Niben101Scf02949g02002	1353	2.0
UAAAAGACUAGGAUUCAAGGU	Niben101Scf00024g02021	3686	2.0
UAAAAGACUAGGAUUCAAGGU	Niben101Scf01752g10014	3015	1.2
UAAAAGACUAGGAUUCAAGGU	Niben101Scf02041g01018	1197	2.0
UGAUGGUGAACUCAUGGAAAUG	Niben101Scf05697g02001	313	2.0

CAAUGAAUUGCAUCAACUUAGG	Niben101Scf00067g02003	350	2.0
CAAUGAAUUGCAUCAACUUAGG	Niben101Scf06789g04019	2037	1.8
UACUUGUUCCUGAACCAGAGCU	Niben101Scf05146g04019	1671	2.0
UGGCUGAUUUCGCUGAAUGCG	Niben101Scf03504g03006	384	1.8
UGGCUGAUUUCGCUGAAUGCG	Niben101Scf05044g02018	65	2.0
UGGCUGAUUUCGCUGAAUGCG	Niben101Scf05044g03009	715	2.0
UGGCUGAUUUCGCUGAAUGCG	Niben101Scf05880g01001	950	2.0
UGACGUUAUUACUUGAAUACC	Niben101Scf09929g01001	2375	2.0
CGGACGAUUCCAUUUAUCUGU	Niben101Scf07069g00003	248	2.0
UGUAACCGGCUUCAGUUUUCUG	Niben101Scf02891g03011	2868	2.0
AAACAGUCGAAGUACAACUACG	Niben101Scf05610g01004	1894	1.5
ACCAGUCAAAGACAAGACUCU	Niben101Scf02105g01013	216	2.0
GAUAGAGGAAUUGUAGUAGACC	Niben101Scf08419g01038	365	2.0
AGGACUGUUAGGCUGCAAGGC	Niben101Scf00288g11016	168	2.0
AGGACUGUUAGGCUGCAAGGC	Niben101Scf00959g00017	483	2.0
AGGACUGUUAGGCUGCAAGGC	Niben101Scf01068g01003	957	2.0
AGGACUGUUAGGCUGCAAGGC	Niben101Scf02362g01007	2112	2.0
AGGACUGUUAGGCUGCAAGGC	Niben101Scf04933g00015	936	1.5
UUGUUUGGGCUUUGAUCAGAGU	Niben101Scf01170g01015	106	1.8
UUGUUUGGGCUUUGAUCAGAGU	Niben101Scf01555g01018	332	2.0
UUGUUUGGGCUUUGAUCAGAGU	Niben101Scf02445g00010	285	2.0
UUGUUUGGGCUUUGAUCAGAGU	Niben101Scf09123g01002	1603	1.8
ACAUGAUCGUCCGCAAUAGACU	Niben101Scf00426g01003	1031	2.0
ACAUGAUCGUCCGCAAUAGACU	Niben101Scf02995g00002	1169	2.0
UUAAGUCUCUUACUGUUGCAGA	Niben101Scf00132g01002	1270	1.8
UUAAGUCUCUUACUGUUGCAGA	Niben101Scf03794g01025	377	2.0
UUAAGUCUCUUACUGUUGCAGA	Niben101Scf06504g01025	908	1.8
UUAAGUCUCUUACUGUUGCAGA	Niben101Scf10015g06004	758	2.0
UCUUUGGAUGAGAAAGACGGAU	Niben101Scf02494g13004	479	2.0
UCUUUGGAUGAGAAAGACGGAU	Niben101Scf07489g01003	409	2.0

UCUUUGGAUGAGAAAGACGGAU	Niben101Scf12528g00007	150	2.0
CUUUGGAAUAGAGCUUUUCU	Niben101Scf00024g00008	476	2.0
CUUUGGAAUAGAGCUUUUCU	Niben101Scf01824g00026	266	2.0
CUUUGGAAUAGAGCUUUUCU	Niben101Scf02921g00004	1512	1.8
CUUUGGAAUAGAGCUUUUCU	Niben101Scf03848g07008	2418	2.0
CUUUGGAAUAGAGCUUUUCU	Niben101Scf06736g08001	3083	1.8
CUUUGGAAUAGAGCUUUUCU	Niben101Scf07227g00010	2423	0.8
CUUUGGAAUAGAGCUUUUCU	Niben101Scf09044g01011	288	2.0
CUUUGGAAUAGAGCUUUUCU	Niben101Scf10712g00036	2416	0.8
CUUUGGAAUAGAGCUUUUCU	Niben101Scf11306g00008	113	2.0
CUUUGGAAUAGAGCUUUUCU	Niben101Scf11884g00021	477	2.0
**PEMV 2 vsiRNAs**			
UUUGGUAGGGGCUGUGCUGCC	Niben101Scf09185g00007.1	3184	1.5
AAGACUGAAACUCAAGCUGAGU	Niben101Scf00861g07009.1	248	1.8
AAGACUGAAACUCAAGCUGAGU	Niben101Scf03634g11004.1	259	1.8
AAGACUGAAACUCAAGCUGAGU	Niben101Scf05822g04008.1	3754	2.0
UGACAAUAAAAGAACUCGAGG	Niben101Scf00764g03003.1	1038	2.0
UUUGGCUCUGAGCUCCAUGGU	Niben101Scf02829g09014.1	311	1.8
UUUGGCUCUGAGCUCCAUGGU	Niben101Scf08127g01015.1	620	2.0
UUUGGCUCUGAGCUCCAUGGU	Niben101Scf08355g03021.1	158	2.0
UUUGGCUCUGAGCUCCAUGGU	Niben101Scf08394g00001.1	611	2.0
UGGAGAAGUUCAACAAUGCUG	Niben101Scf03400g02010.1	490	1.8
UGGAGAAGUUCAACAAUGCUG	Niben101Scf08991g02002.1	589	2.0
AAGGGAGCGAUGUCGAACAGG	Niben101Scf02982g03019.1	536	2.0
AAGGGAGCGAUGUCGAACAGG	Niben101Scf10608g03010.1	699	2.0
UUGUCCGUGUAGAACACCCUU	Niben101Scf03739g07009.1	873	2.0
AAGGUCAAAGGUAGACGCAUG	Niben101Scf00863g17026.1	1184	2.0
CAUACUGAUCUCUAUAAAU	Niben101Scf00051g04005.1	700	1.5
CAUACUGAUCUCUAUAAAU	Niben101Scf00181g00004.1	4190	2.0
CAUACUGAUCUCUAUAAAU	Niben101Scf00863g02001.1	4199	2.0

CAUACUGAUCUCUAUAAAU	Niben101Scf00955g04005.1	290	2.0
CAUACUGAUCUCUAUAAAU	Niben101Scf02321g00027.1	299	2.0
CAUACUGAUCUCUAUAAAU	Niben101Scf05750g04002.1	1957	2.0
CAUACUGAUCUCUAUAAAU	Niben101Scf19230g01024.1	1897	2.0
UUGUGGGUCACUGCUUCGAUG	Niben101Scf16556g00009.1	1989	2.0
CCAAUUUGCAGAACGAUGGU	Niben101Scf12372g02004.1	2137	1.8
AAGACUGAAACUCAAGCUGAA	Niben101Scf00861g07009.1	248	1.0
AAGACUGAAACUCAAGCUGAA	Niben101Scf03634g11004.1	259	1.0
AAGACUGAAACUCAAGCUGAA	Niben101Scf05822g04008.1	3754	1.0
AGCAUUGUUGAACUUCUCCAAG	Niben101Scf01940g00011.1	283	2.0
AGCAUUGUUGAACUUCUCCAAG	Niben101Scf09028g00008.1	269	1.5
UGACAUGGACACCUCUUUGGG	Niben101Scf09928g01020.1	109	2.0
CAUUGUCCGUGUAGAACACC	Niben101Scf00854g07045.1	373	2.0
UUAGCAACUGCAGGGUACUUGU	Niben101Scf02511g06004.1	2186	2.0
UGCGUCCUCUUGGAGAUUUUG	Niben101Scf00270g13017.1	270	2.0
UGCGUCCUCUUGGAGAUUUUG	Niben101Scf02148g00017.1	1284	1.2
UGCGUCCUCUUGGAGAUUUUG	Niben101Scf03722g00008.1	1738	1.8
ACAUGAGAAACAGGCUGAAUG	Niben101Scf08783g00010.1	1723	2.0
UGGAGCCGAUGCUGUACAAAGU	Niben101Scf02575g00005.1	527	2.0
CAAUUUGCAGAACGAUGGUGG	Niben101Scf12372g02004.1	2136	1.5
AAUUUGGCUCUGAGCUCCAUGG	Niben101Scf02829g09014.1	313	1.8
AAUUUGGCUCUGAGCUCCAUGG	Niben101Scf08355g03021.1	160	2.0
UGAGACGGGGGAGAUCAUUGC	Niben101Scf04283g10001.1	299	2.0
UGAGACGGGGGAGAUCAUUGC	Niben101Scf07577g00007.1	1964	1.8
GUUUGGCAGAACUCUAUUUUC	Niben101Scf02359g00008.1	846	2.0
UCCUGCGGGAUAACUGUGUC	Niben101Scf03819g03027.1	706	2.0
UCCUGCGGGAUAACUGUGUC	Niben101Scf10616g01013.1	707	2.0
AAGACUGAAACUCAAGCUGAAC	Niben101Scf00861g07009.1	248	1.5
AAGACUGAAACUCAAGCUGAAC	Niben101Scf03634g11004.1	259	1.5
GGUUGAACCGCCGGUUGACGU	Niben101Scf02915g00007.1	50	1.5

AAAUGGUUCUGUGGGUGCCUCU	Niben101Ctg15720g00002.1	1358	2.0
AAAUGGUUCUGUGGGUGCCUCU	Niben101Scf07590g05006.1	1666	2.0
UUGCUGUUGGGAUGCCACCUC	Niben101Scf02635g05003.1	547	2.0
CAACUGUGUGCUCAUGGUGCU	Niben101Scf01056g03008.1	650	1.8
AAUAGAGUUCUGCCAAACGCA	Niben101Scf01837g02002.1	1558	2.0
AAUAGAGUUCUGCCAAACGCA	Niben101Scf03422g02008.1	2325	2.0
AAUAGAGUUCUGCCAAACGCA	Niben101Scf09264g00009.1	2415	2.0
AAUAGAGUUCUGCCAAACGCA	Niben101Scf11980g01002.1	1667	2.0
CCAAGACUGAAACUCAAGC	Niben101Scf00821g08003.1	2461	2.0
CCAAGACUGAAACUCAAGC	Niben101Scf00861g07009.1	250	1.5
CCAAGACUGAAACUCAAGC	Niben101Scf01795g02012.1	162	2.0
CCAAGACUGAAACUCAAGC	Niben101Scf01849g03012.1	9353	2.0
CCAAGACUGAAACUCAAGC	Niben101Scf03634g11004.1	261	1.5
CCAAGACUGAAACUCAAGC	Niben101Scf04400g02005.1	579	2.0
CCAAGACUGAAACUCAAGC	Niben101Scf04594g02003.1	503	2.0
CCAAGACUGAAACUCAAGC	Niben101Scf15381g00010.1	195	2.0
CCAAGACUGAAACUCAAGC	Niben101Scf15963g01011.1	697	2.0
UCAUCCGUUAUUAGCUGGGACU	Niben101Scf01991g06001.1	1036	1.8
UCAUCCGUUAUUAGCUGGGACU	Niben101Scf25279g00004.1	1027	1.8
UGCAAAUUGGCAGAGCACGUG	Niben101Scf02887g01014.1	197	2.0
UUGUCCUGGCUAAAUACAUCA	Niben101Ctg11091g00003.1	588	2.0
CGAGCUUUUCAACAAUGGUGA	Niben101Scf01399g00014.1	2076	2.0
CGAGCUUUUCAACAAUGGUGA	Niben101Scf03018g03003.1	622	1.8
CAAAGAGACAUCACUGUAGGC	Niben101Scf01764g02020.1	1652	2.0
CCUCAGGAUAGUGAGCUCC	Niben101Scf00404g03003.1	1159	1.5
CCUCAGGAUAGUGAGCUCC	Niben101Scf04879g00002.1	538	2.0

Supplementary Tabe S3 GO analysis of the predicted target genes of vsiRNAs

Term type	GO accession	Description	Number of genes
**BrYV**			
biological process	GO:0008152	metabolic process	166
biological process	GO:0071704	organic substance metabolic process	141
biological process	GO:0044238	primary metabolic process	136
biological process	GO:0044699	single-organism process	85
biological process	GO:0044763	single-organism cellular process	81
biological process	GO:1901360	organic cyclic compound metabolic process	66
biological process	GO:0006725	cellular aromatic compound metabolic process	62
biological process	GO:0006807	nitrogen compound metabolic process	61
biological process	GO:0044710	single-organism metabolic process	60
biological process	GO:0034641	cellular nitrogen compound metabolic process	59
biological process	GO:0046483	heterocycle metabolic process	59
biological process	GO:0006139	nucleobase-containing compound metabolic process	54
biological process	GO:0090304	nucleic acid metabolic process	44
biological process	GO:0050789	regulation of biological process	42
biological process	GO:0065007	biological regulation	41
biological process	GO:1901362	organic cyclic compound biosynthetic process	40
biological process	GO:0050794	regulation of cellular process	38
biological process	GO:0006796	phosphate-containing compound metabolic process	34
biological process	GO:0006793	phosphorus metabolic process	33
biological process	GO:0018130	heterocycle biosynthetic process	32
biological process	GO:0019438	aromatic compound biosynthetic process	32
biological process	GO:0050896	response to stimulus	31
biological process	GO:0080090	regulation of primary metabolic process	28
biological process	GO:0009889	regulation of biosynthetic process	26
biological process	GO:0010556	regulation of macromolecule biosynthetic process	26

biological process	GO:0043412	macromolecule modification	26
biological process	GO:0006355	regulation of transcription, DNA-dependent	24
biological process	GO:0006464	cellular protein modification process	24
biological process	GO:0051171	regulation of nitrogen compound metabolic process	24
biological process	GO:0051252	regulation of RNA metabolic process	23
biological process	GO:2001141	regulation of RNA biosynthetic process	23
biological process	GO:0036211	protein modification process	21
biological process	GO:0019222	regulation of metabolic process	20
biological process	GO:0019219	regulation of nucleobase-containing compound metabolic process	19
biological process	GO:0031323	regulation of cellular metabolic process	19
biological process	GO:1901564	organonitrogen compound metabolic process	18
biological process	GO:2000112	regulation of cellular macromolecule biosynthetic process	17
biological process	GO:0016310	phosphorylation	16
biological process	GO:0051716	cellular response to stimulus	15
biological process	GO:0031326	regulation of cellular biosynthetic process	14
biological process	GO:0060255	regulation of macromolecule metabolic process	14
biological process	GO:0006468	protein phosphorylation	11
biological process	GO:0007154	cell communication	11
biological process	GO:0023052	signaling	10
biological process	GO:0044700	single organism signaling	8
biological process	GO:0006629	lipid metabolic process	7
biological process	GO:0007165	signal transduction	7
cellular component	GO:0005634	nucleus	24
cellular component	GO:0044422	organelle part	17
cellular component	GO:0044446	intracellular organelle part	17
molecular function	GO:0003674	molecular function	274
molecular function	GO:0005488	binding	190
molecular function	GO:0003824	catalytic activity	175
molecular function	GO:0043167	ion binding	121
molecular function	GO:1901363	heterocyclic compound binding	113

molecular function	GO:0097159	organic cyclic compound binding	108
molecular function	GO:0016740	transferase activity	69
molecular function	GO:0005515	protein binding	68
molecular function	GO:0043168	anion binding	68
molecular function	GO:0000166	nucleotide binding	66
molecular function	GO:1901265	nucleoside phosphate binding	64
molecular function	GO:0036094	small molecule binding	60
molecular function	GO:0001883	purine nucleoside binding	58
molecular function	GO:0032553	ribonucleotide binding	57
molecular function	GO:0017076	purine nucleotide binding	53
molecular function	GO:0032555	purine ribonucleotide binding	53
molecular function	GO:0032559	adenyl ribonucleotide binding	51
molecular function	GO:0032549	ribonucleoside binding	50
molecular function	GO:0032550	purine ribonucleoside binding	50
molecular function	GO:0030554	adenyl nucleotide binding	48
molecular function	GO:0035639	purine ribonucleoside triphosphate binding	48
molecular function	GO:0001882	nucleoside binding	47
molecular function	GO:0005524	ATP binding	47
molecular function	GO:0016772	transferase activity, transferring phosphorus-containing groups	43
molecular function	GO:0016301	kinase activity	28
molecular function	GO:0016773	phosphotransferase activity, alcohol group as acceptor	18
molecular function	GO:0004672	protein kinase activity	14
molecular function	GO:0016817	hydrolase activity, acting on acid anhydrides	14
molecular function	GO:0016818	hydrolase activity, acting on acid anhydrides, in phosphorus-containing anhydrides	12
molecular function	GO:0016462	pyrophosphatase activity	11
molecular function	GO:0017111	nucleoside-triphosphatase activity	6
**PEMV 2**			
biological process	GO:0008150	biological process	234
biological process	GO:0009987	cellular process	187

biological process	GO:0008152	metabolic process	181
biological process	GO:0071704	organic substance metabolic process	147
biological process	GO:0044237	cellular metabolic process	145
biological process	GO:0044238	primary metabolic process	130
biological process	GO:0043170	macromolecule metabolic process	111
biological process	GO:0044260	cellular macromolecule metabolic process	97
biological process	GO:0009058	biosynthetic process	81
biological process	GO:0044763	single-organism cellular process	76
biological process	GO:0044699	single-organism process	73
biological process	GO:1901360	organic cyclic compound metabolic process	72
biological process	GO:0006807	nitrogen compound metabolic process	71
biological process	GO:0044249	cellular biosynthetic process	71
biological process	GO:1901576	organic substance biosynthetic process	70
biological process	GO:0046483	heterocycle metabolic process	68
biological process	GO:0006725	cellular aromatic compound metabolic process	64
biological process	GO:0034641	cellular nitrogen compound metabolic process	64
biological process	GO:0006139	nucleobase-containing compound metabolic process	57
biological process	GO:0044710	single-organism metabolic process	57
biological process	GO:0090304	nucleic acid metabolic process	53
biological process	GO:0019538	protein metabolic process	51
biological process	GO:0051179	localization	50
biological process	GO:0065007	biological regulation	46
biological process	GO:0050789	regulation of biological process	45
biological process	GO:1901362	organic cyclic compound biosynthetic process	42
biological process	GO:0019438	aromatic compound biosynthetic process	41
biological process	GO:0051234	establishment of localization	41
biological process	GO:0006810	transport	40
biological process	GO:0016070	RNA metabolic process	40
biological process	GO:0044267	cellular protein metabolic process	40
biological process	GO:0018130	heterocycle biosynthetic process	39

biological process	GO:0050794	regulation of cellular process	38
biological process	GO:0034654	nucleobase-containing compound biosynthetic process	31
biological process	GO:0044271	cellular nitrogen compound biosynthetic process	30
biological process	GO:0031323	regulation of cellular metabolic process	29
biological process	GO:0019222	regulation of metabolic process	27
biological process	GO:0051171	regulation of nitrogen compound metabolic process	27
biological process	GO:0006355	regulation of transcription, DNA-dependent	26
biological process	GO:0060255	regulation of macromolecule metabolic process	26
biological process	GO:0080090	regulation of primary metabolic process	26
biological process	GO:0009889	regulation of biosynthetic process	25
biological process	GO:0010468	regulation of gene expression	25
biological process	GO:0010556	regulation of macromolecule biosynthetic process	25
biological process	GO:0044765	single-organism transport	25
biological process	GO:2001141	regulation of RNA biosynthetic process	24
biological process	GO:0032774	RNA biosynthetic process	23
biological process	GO:0006351	transcription, DNA-dependent	22
biological process	GO:0006464	cellular protein modification process	22
biological process	GO:0050896	response to stimulus	21
biological process	GO:0006796	phosphate-containing compound metabolic process	20
biological process	GO:0051252	regulation of RNA metabolic process	20
biological process	GO:0019219	regulation of nucleobase-containing compound metabolic process	17
biological process	GO:2000112	regulation of cellular macromolecule biosynthetic process	16
biological process	GO:0006793	phosphorus metabolic process	15
biological process	GO:0031326	regulation of cellular biosynthetic process	15
biological process	GO:0043412	macromolecule modification	15
biological process	GO:1901564	organonitrogen compound metabolic process	15
biological process	GO:0051716	cellular response to stimulus	13
biological process	GO:0016310	phosphorylation	12
biological process	GO:0036211	protein modification process	12
biological process	GO:0006629	lipid metabolic process	9

biological process	GO:0005975	carbohydrate metabolic process	8
biological process	GO:0044281	small molecule metabolic process	8
biological process	GO:0006468	protein phosphorylation	7
biological process	GO:0071702	organic substance transport	7
biological process	GO:0007154	cell communication	5
biological process	GO:0009056	catabolic process	5
biological process	GO:0051641	cellular localization	5
biological process	GO:0007165	signal transduction	4
biological process	GO:0023052	signaling	4
biological process	GO:0044700	single organism signaling	4
biological process	GO:0051649	establishment of localization in cell	2
biological process	GO:1901135	carbohydrate derivative metabolic process	2
biological process	GO:1901566	organonitrogen compound biosynthetic process	1
cellular component	GO:0005575	cellular component	148
cellular component	GO:0016020	membrane	81
cellular component	GO:0044425	membrane part	46
cellular component	GO:0043231	intracellular membrane-bounded organelle	35
cellular component	GO:0031224	intrinsic to membrane	29
cellular component	GO:0043227	membrane-bounded organelle	28
cellular component	GO:0016021	integral to membrane	14
cellular component	GO:0005634	nucleus	13
molecular function	GO:0003674	molecular_function	295
molecular function	GO:0005488	binding	194
molecular function	GO:0003824	catalytic activity	179
molecular function	GO:0097159	organic cyclic compound binding	103
molecular function	GO:1901363	heterocyclic compound binding	103
molecular function	GO:0043167	ion binding	81
molecular function	GO:0005515	protein binding	68
molecular function	GO:0003676	nucleic acid binding	54
molecular function	GO:0016740	transferase activity	54

molecular function	GO:0043168	anion binding	50
molecular function	GO:0000166	nucleotide binding	48
molecular function	GO:0016787	hydrolase activity	48
molecular function	GO:0017076	purine nucleotide binding	46
molecular function	GO:0001882	nucleoside binding	45
molecular function	GO:0032550	purine ribonucleoside binding	45
molecular function	GO:0036094	small molecule binding	45
molecular function	GO:0032549	ribonucleoside binding	44
molecular function	GO:1901265	nucleoside phosphate binding	44
molecular function	GO:0032553	ribonucleotide binding	43
molecular function	GO:0001883	purine nucleoside binding	42
molecular function	GO:0032555	purine ribonucleotide binding	41
molecular function	GO:0035639	purine ribonucleoside triphosphate binding	37
molecular function	GO:0030554	adenyl nucleotide binding	36
molecular function	GO:0032559	adenyl ribonucleotide binding	36
molecular function	GO:0005524	ATP binding	33
molecular function	GO:0003677	DNA binding	29
molecular function	GO:0016772	transferase activity, transferring phosphorus-containing groups	22
molecular function	GO:0016301	kinase activity	17
molecular function	GO:0016773	phosphotransferase activity, alcohol group as acceptor	12
molecular function	GO:0004672	protein kinase activity	8
molecular function	GO:0016462	pyrophosphatase activity	8
molecular function	GO:0017111	nucleoside-triphosphatase activity	7
molecular function	GO:0016817	hydrolase activity, acting on acid anhydrides	6
molecular function	GO:0016818	hydrolase activity, acting on acid anhydrides, in phosphorus-containing anhydrides	5

Supplementary Tabe S4 Primers used in this study

Primer	Sequence (5' - 3')
PEM2-001F	GGGTATTTATAGAGATC
PEM2-Bg4253R	GA*AGATCT*[1]GGGCGCCAGGGAGGTAA
BrA-P3-EcoRF	*GAATTC*ATGAATACGGTCG
BrA-P3-HindR	*AAGCTT*CTATTTGGGATTATGG
Br-001F	ACAAAAGAAACCAGGTGGGAATCCTAAGTTGATGCAATTC
Br-241F	CATCCACGATGATGTCAGGAAGTCCATACTTGTTCCTGAA
Br-741F	TCCTTTTCAGCTCTTCGGTCTACACCGAAATGTTTGTATG
Br-1241F	CTCCGGAAGCCCCTACTTTAATGGTAAAACCATTCTAGGG
Br-1741F	GAAGAGCTTCAAGGAAACAGCCGGAGAGTTCGAAAGATAT
Br-2241F	ACCGTGGGTGGGTAGAAGATCCAAAGCTCC TCCCAGTCCT
Br-2741F	GACCTCAACCCTGCAACAGAAAGATTAAGATCCTGTTGGT
Br-3241F	GCTACCACAAAAGATATCCAGGGAGTAAAGAAGCAAACAA
Br-3741F	CTGCCCGGCATTCTCTAATGGAATACTCAAGGCCTACCAT
Br-4241F	GCCTCCGGTACATTGAGAATGAAAACATGAACTGGACCAA
Br-4741F	TCTGTTTCTTTGGATGAGAAAGACGGATCAAGAGGTTCCG
Br-5241F	GGTTACACCGGACGAGTATGAGCAGGCGAAGCTGAAAGAT
Br-5541F	CTTATCTAGCTTTGTTAATATTTAGCTAATAAAGTCAAGC
Br-40R	GAATTGCATCAACTTAGGATTCCCACCTGGTTTCTTTTGT
Br-280R	TTCAGGAACAAGTATGGACTTCCTGACATCATCGTGGATG
Br-780R	CATACAAACATTTCGGTGTAGACCGAAGAGCTGAAAAGGA
Br-1280R	CCCTAGAATGGTTTTACCATTAAAGTAGGGGCTTCCGGAG
Br-1780R	ATATCTTTCGAACTCTCCGGCTGTTTCCTTGAAGCTCTTC
Br-2280R	AGGACTGGGAGGAGCTTTGGATCTTCTACCCACCCACGGT
Br-2780R	ACCAACAGGATCTTAATCTTTCTGTTGCAGGGTTGAGGTC
Br-3280R	TTGTTTGCTTCTTTACTCCCTGGATATCTTTTGTGGTAGC
Br-3780R	ATGGTAGGCCTTGAGTATTCCATTAGAGAATGCCGGGCAG
Br-4280R	TTGGTCCAGTTCATGTTTTCATTCTCAATGTACCGGAGGC

Br-4780R	CGGAACCTCTTGATCCGTCTTTCTCATCCAAAGAAACAGA
Br-5280R	ATCTTTCAGCTTCGCCTGCTCATACTCGTCCGGTGTAACC
Br-5580R	GCTTGACTTTATTAGCTAAATATTAACAAAGCTAGATAAG
PE-001F	GGGTATTTATAGAGATCAGTATGAACTGTGTCGCTAGAAT
PE-241F	TATTTGGCCCCTCCATCGCCCCTGAGCCTGTGGCTCTAGT
PE-741F	TTTGGCGTCTGCAAACGCACCCCCGCAAACTTAGAGATGG
PE-1241F	GAGAGTTACACTGGGTCCCAGCGCACTCGCTATGCCAACG
PE-1741F	TTACAAGGTCAAAGGTAGACGCATGAGCGGTGACATGGAC
PE-2241F	ACGAGGGGTTGAATTGGTACAGGATGGGAATGGACCTTTC
PE-2741F	TTCGGCCCGCCAGCCTAGGACAATGGCGGATGGGTAATAT
PE-3241F	GCCACCGAGCTGTTCGAAAGCAACTTCGCACCGGGGTGCA
PE-3741F	ACGCAGGTGTCGGAGGGATCGCCAGCCCTCAGGATAGTGA
PE-4141F	AGGCTCGTCGTAATTAGTTGCAGCGACGTTAATCAACCCG
PE-40R	ATTCTAGCGACACAGTTCATACTGATCTCTATAAATACCC
PE-280R	ACTAGAGCCACAGGCTCAGGGGCGATGGAGGGGCCAAATA
PE-780R	CCATCTCTAAGTTTGCGGGGGTGCGTTTGCAGACGCCAAA
PE-1280R	CGTTGGCATAGCGAGTGCGCTGGGACCCAGTGTAACTCTC
PE-1780R	GTCCATGTCACCGCTCATGCGTCTACCTTTGACCTTGTAA
PE-2280R	GAAAGGTCCATTCCCATCCTGTACCAATTCAACCCCTCGT
PE-2780R	ATATTACCCATCCGCCATTGTCCTAGGCTGGCGGGCCGAA
PE-3280R	TGCACCCCGGTGCGAAGTTGCTTTCGAACAGCTCGGTGGC
PE-3780R	TCACTATCCTGAGGGCTGGCGATCCCTCCGACACCTGCGT
PE-4180R	CGGGTTGATTAACGTCGCTGCAACTAATTACGACGAGCCT

[1] Restriction sites are highlighted with italic.
